# Ammonium release in synthetic and human urine by a urease immobilized nanoconstruct†

**DOI:** 10.1039/d3ra07606g

**Published:** 2024-02-27

**Authors:** Manab Diasi, Rinki Singh, Amarjyoti Das Mahapatra, Renuka L, Hitarth Patel, Hasit Ganatra, Bhaskar Datta

**Affiliations:** a Department of Chemistry, Indian Institute of Technology Gandhinagar Palaj Gandhinagar 382355 Gujarat India bdatta@iitgn.ac.in rinkoosingh62@gmail.com; b Blasto Research Private Limited Ahmedabad Gujarat India; c Department of Biological Engineering, Indian Institute of Technology Gandhinagar Palaj Gandhinagar 382355 Gujarat India

## Abstract

In this work, we have studied the ability of urease immobilized on glutaraldehyde crosslinked chitosan coated magnetic iron oxide nanoparticles (Urease/GA/CS/MIONPs), for the hitherto unreported comparative hydrolysis of urea in synthetic (SUr) and real human urine (HUr). The prepared Urease/GA/CS/MIONPs were characterized by a combination of Fourier transform infrared spectroscopy (FTIR), field emission-scanning-electron-microscopy (FESEM), energy dispersive X-ray spectroscopy (EDX) and dynamic light scattering (DLS). The nanoconstructs display the highest ammonium ion liberation post-urea hydrolysis in 1/20 or 1/24-fold dilutions of SUr and HUr, respectively. The optimum activity of immobilized urease is observed at pH 7, and the nanoconstructs facilitate efficient urea-hydrolysis till at least 45 °C. Kinetic analysis of the immobilized urease shows *k*_m_ and *v*_max_ of 14.81 mM, 12.36 mM, and 18.55 μM min^−1^ and 10.10 μM min^−1^, towards SUr and HUr, respectively. The magnetization of the immobilized urease is suitable for reuse across multiple cycles of urea hydrolysis in SUr and HUr. The robust performance of Urease/GA/CS/MIONPs in SUr and HUr is promising for generating ammonium as a useable source of nitrogen from human urine, and underscores the suitability of SUr as a urine mimic for such interventions.

## Abstract 2

Urease nanoconstructs are capable of releasing similar levels of ammonium in diluted synthetic and real human urine.

## Graphical abstract

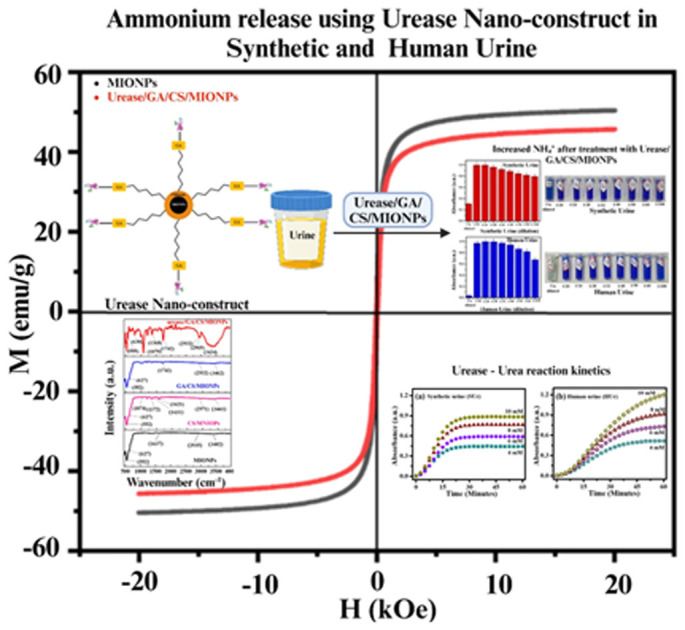

## 1 Introduction

Nitrogen in the earth's atmosphere is benign and unusable unless it is actively fixed in a useable form. The Haber–Bosch process revolutionized the ability to fix nitrogen into ammonia.^1,2^ Ammonia or ammonium ions are key components of fertilizers. Ammonia serves as the most prominent source of useable and active nitrogen species. Ammonia and carbon dioxide are used to produce urea, a nitrogen dense compound that has expansive industrial usage.^1,3^ While wastewater and waste streams are rich in nitrogen, the production of useable nitrogen continues to rely on the energy-intensive Haber–Bosch process.^1^ The push towards sustainable living in the current century includes the search for nitrogen fixation processes that place a significantly lower burden. Human urine is an attractive candidate for use in nitrogen harvesting.^4^ Technology enabling the use of human waste as a fertilizer is yet to attain maturity and human urine is seldom included in the discourse on commercially viable fertilizers.^5^ Each person excretes 0.8 to 1.3 l of urine every day, containing 9 to 23 g L^−1^ of urea (H_2_NCONH_2_). Urine contributes roughly 75–80% of the total nitrogen, apart from 50% of the total phosphorus loading in sewage, even though it accounts for just 1% of the sewage flow.^6^ Urine is a minor component of waste water albeit arguably the most attractive candidate for nitrogen harvesting. The recovery of nitrogen from urine is likely to bring us closer to a circular economy and contribute to sustainable living practices.^7,8^ The physico-chemical characteristics of urine create unique challenges in nitrogen harvesting especially considering the differences in behaviour of fresh *versus* stored urine. When the body first excretes urine, the nitrogen is in the form of urea and the urine is labelled as being “fresh”. The urea undergoes hydrolysis over time upon coming into contact with the enzyme urease.^9,10^ Hydrolysis of urea produces ammonia and bicarbonate and “hydrolyzed urine” is labelled as the form of urine where nitrogen is chiefly present as ammonia.^11^ A limited number of approaches are reported for nitrogen recovery from human urine. The reported strategies are based on the conversion of urea to ammonia at high pH achieved by storage and subsequent alkali addition, followed by stripping *via* the addition of strong acids.^12^ Other approaches include biological nitrification using a hybrid membrane-aerated biofilm reactor followed by distillation.^13^

The eco-cycling of nutrients from source separated urine has been appreciated for reducing eutrophication in freshwater bodies. Urine separating toilets have been developed and installed in eco-villages around the world. These units collect urine separately from faeces for further use as fertilizer.^14,15^ While life-cycle assessment studies of urine-separation systems indicate advantageous nitrogen and phosphorus-recycling efficiency, they also show that the storage, transport, and spreading of large amounts of urine pose serious obstacles to the efficiency of use. Large volumes of urine are needed to fertilize agricultural land, and in addition to high transportation costs, large volumes suffer from losses from ammonia evaporation.^16,17^

The enzyme urease is produced by a wide variety of bacteria, fungi, and plants and catalyzes the hydrolysis of urea to ammonia.^18^ Urease has been extensively studied in the contexts of soil science and human gastric and urinary health. However, remarkably little has been reported regarding the planned use of urease in a urine treatment paradigm.^19^ Issues pertaining to stability, cost, and reusability of enzymes could be a reason for the dearth of studies on urease for ammonia harvesting.^20–22^ The immobilization of enzymes on solid supports such as polymeric gels and membranes, silica, and zeolites have been used to address enzyme stability and reusability and expand the scope of application across a range of process parameters.^23–26^ Magnetic iron oxide nanoparticles have been widely used for immobilization and efficient use of enzymes. The ability to separate such nanoconstructs using an external magnetic field is a distinctive aspect of these carriers.^27–29^ We have previously observed the singular behaviour of hydrolytic enzymes immobilized on magnetic iron oxide nanoparticles in generating unique products of hydrolysis from food waste.^30^

In this work, we have investigated the use of urease-immobilized magnetic iron oxide nanoparticles for the hydrolysis of synthetic and fresh real human urine. While immobilized urease has been examined for its ability to hydrolyze urea, there is a notable dearth of reports that compare the performance of such constructs across synthetic and real human urine. We have assessed the ammonia released upon hydrolysis of synthetic and real human urine under conditions of dilution. We observe comparable hydrolytic behaviour of immobilized urease towards synthetic and real human urine in terms of the reaction conditions including dilution, pH, temperature, and reusability of constructs. Immobilized urease facilitates the production of significantly higher amounts of ammonia in both diluted synthetic and real urine, in contrast to undiluted samples, and affords attractive reusability. Our results point to the suitability of synthetic urine as a model system for improving the performance of immobilized urease. This work projects urease-immobilized magnetic iron oxide as an attractive enzyme construct for treating urine under ambient conditions facilitating subsequent harvesting of ammonia.

## 2. Experimental section

### 2.1. Materials

Urease (Jack bean *Canavalia ensiformis*), glutaraldehyde solution (Grade II, 25% in H_2_O), and Chitosan were purchased from Sigma Aldrich. For Fe_3_O_4,_ magnetic nanoparticle synthesis, iron(ii) chloride (FeCl_2_) (Merck) and iron(iii) chloride (FeCl_3_) (Merck) were used. Cetyltrimethylammonium bromide (CTAB) (Merck) was used as a surfactant. Synthetic urine (SUr) was prepared as reported previously.^31^ Sodium sulphate (Na_2_SO_4_), uric acid (C_5_H_4_N_4_O_3_), sodium citrate (Na_3_C_6_H_5_O_7_·2H_2_O), creatinine (C_4_H_7_N_3_O), urea (CH_4_N_2_O), potassium chloride (KCl), sodium chloride (NaCl), calcium chloride (CaCl_2_), ammonium chloride (NH_4_Cl), potassium oxalate (K_2_C_2_O_4_·H_2_O), magnesium sulphate (MgSO_4_·7H_2_O), sodium phosphate monobasic monohydrate (NaH_2_PO_4_·2H_2_O), sodium phosphate dibasic dihydrate (Na_2_HPO_4_·2H_2_O) were used and were purchased from Sigma Aldrich, Bengaluru, India. A urea estimation kit in urine (based on the Berthelot method) was procured from Labcare Diagnostics, Gujarat, India, and used based on the vendor instructions. Fresh real human urine was collected by participating volunteers in plastic collection vessels, without dilution, and stored in bottles to be utilized within 24 h. All experiments with real human urine were performed within 24 h of collection. The use of real human urine for this work was exempted from review by the Institutional Ethics Committee of the Indian Institute of Technology Gandhinagar (Institutional Ethics Committee ID no. IEC/2022-2023/EXM/BD/001). Informed consent was obtained from all volunteers who contributed human urine to this work. For the Berthelot reaction, sodium hydroxide (NaOH), phenol, sodium nitroprusside, and sodium hypochlorite (NaOCl) were purchased from Sigma Aldrich, Bengaluru, India. All aqueous solutions were prepared with deionized water.

### 2.2. Synthesis of magnetic iron-oxide nanoparticles (MIONPs)

**Fig. 1:**
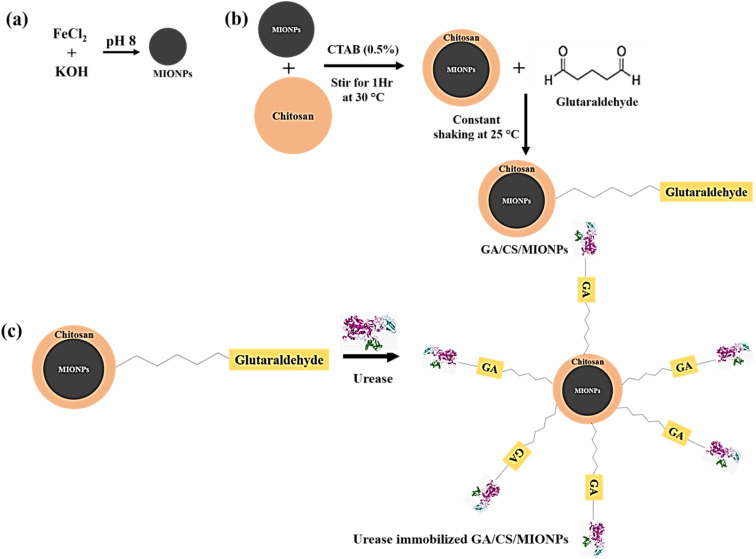
Schematic depicting the synthesis of (a) magnetic iron-oxide nanoparticles (MIONPs), (b) glutaraldehyde crosslinked chitosan-modified magnetic iron-oxide nanoparticles (GA/CS/MIONPs), and (c) urease-immobilized glutaraldehyde crosslinked chitosan-modified magnetic iron-oxide nanoparticles (Urease/GA/CS/MIONPs). The representations of the constructs and intermediates are not proportional in scale.

Magnetic iron oxide nanoparticles (MIONPs), Fe_3_O_4_ were prepared by the alkaline hydrolysis of ferrous ions, as previously reported.^30^ A potassium hydroxide (KOH, 1 M) solution was added dropwise into a 50 mL solution of FeCl_2_ (0.05 M) while being continuously stirred until the pH reached 8. The solution was further kept for precipitation for 2 h. A vacuum filter was used to separate the black coloured precipitate, washed twice with ultrapure water and two times with ethanol, followed by drying, and storage in a vacuum desiccator. Schematic for the synthesis of MIONPs is shown in Fig. 1a.

### 2.3. Immobilization of urease on MIONPs

#### 2.3.1 Chitosan coating on MIONPs

To synthesize modified magnetic nanoparticles, a chitosan (CS) solution was applied to the surface of MIONPs as shown in Fig. 1b. Briefly, 2 g of CTAB were dissolved in 400 mL of deionized water and used to disperse 0.25 g of magnetic nanoparticles. 100 mL of chitosan solution (0.02 gram CS powder mixed in 100 mL of 1% (w/v) acetic acid solution) was then gradually added to the aforementioned solution. The mixture was centrifuged at 1000 rpm for 1 h at room temperature. The CS coated MIONPs were then carefully washed with ethanol and deionized water (1 : 1) several times before being magnetically separated from the solution using a permanent neodymium magnet. The resulting chitosan modified nanoparticles were allowed to dry overnight at 60 °C.^32^

#### 2.3.2 Glutaraldehyde mediated immobilization of urease on chitosan coated MIONPs

40 mg of chitosan modified magnetic nanoparticles were taken in 2.5% glutaraldehyde (8 mL) and further sonicated for 10 min at room temperature.^33,34^ The suspension was gently shaken for one hour at room temperature. The particles were then subjected to two cycles of magnetic decantation and washed with pH 7 buffer (PBS) before being weighed to determine yield. After adding 2 mL of urease enzyme solution (4 mg mL^−1^), the mixture was stirred for 24 hours at room temperature. The suspension was directly used in subsequent experiments (Fig. 1c).

### 2.4. Characterization of urease-immobilized chitosan-coated MIONPs

ATR-FTIR of magnetic iron oxide nanoparticles (MIONPs), chitosan coated MIONPs (CS/MIONPs), glutaraldehyde crosslinked chitosan coated MIONPs (GA/CS/MIONPs) and urease immobilized chitosan coated MIONPs (Urease/GA/CS/MIONPs) were obtained with a Fourier transform infrared spectrometer (PerkinElmer Spectrum Two) in the range of 4000–400 cm^−1^. The structural properties and crystallinity of MIONPs (Fe_3_O_4_), CS/MIONPs, GA/CS/MIONPs were investigated by X-ray diffraction (XRD) using a Rigaku SmartLab automated multipurpose X-ray diffractometer using Cu Kα radiation (*λ* = 1.54 Å). The surface morphology of the MIONPs (Fe_3_O_4_), GA/CS/MIONPs and Urease/GA/CS/MIONPs was explored by Field emission scanning electron microscopy (FE-SEM) on a JEOL JSM 7600F (USA). Dynamic light scattering (DLS) of MIONPs (Fe_3_O_4_), GA/CS/MIONPs and Urease/GA/CS/MIONPs were performed to obtain hydrodynamic diameter using NanoZS Malvern UK instrument. The spectrophotometric measurements were carried out using UV–Visible spectrophotometer (Analytic Jena Specord 210 Plus) with a spectral range of 200–800 nm. The magnetization of MIONPs (Fe_3_O_4_) and the Urease/GA/CS/MIONPs were measured at room temperature (25 °C) in a magnetic field varying from −2 to +2 T using a PPMS-9T vibration sample magnetometer.

### 2.5. Immobilized urease-catalyzed hydrolysis of synthetic and human urine

#### 2.5.1 Effects of dilution of urine

The urease-catalyzed hydrolysis of urea present in synthetic or real human urine was assessed by use of Berthelot reaction.^35^ Briefly, urease nanoconstruct was taken in a quartz cuvette (50 μL 0.1 mg mL^−1^) and the subsequent additions were performed in a heating bath at 25 °C. Two reagents, reagent A and B, for the assay of urease – catalyzed hydrolysis of urea were prepared as follows: reagent A was prepared by dissolving phenol (50 mg, 5.3 mmol) and sodium nitroprusside (25 mg, 0.084 mmol) in 5 mL of DI water, while reagent B was prepared by dissolving NaOH (25 mg, 0.00625 mmol) and NaOCl which contains 5% active chlorine (52.5 μL) in 5 mL of DI water. 857 μL of reactant A, 25 μL of four different dilutions (undiluted, 1/20, 1/24, 1/32, 1/40, 1/60 and 1/100) of synthetic and real fresh urine, respectively, and 857 μL of reactant B were added to the cuvette. Indophenol blue dye formation was initiated immediately after addition of reactant B. UV-visible spectrophotometer was used to measure the indophenol dye's absorbance at 635 nm, and the absorbance was monitored over a period of 1 h. The measured absorbance is directly proportional to the concentration of ammonia present at a specific time point in the solution.^31,36^ The standard errors of measurement of ammonia in these experiments were in the range of 1–3% without urease treatment, and 1–8% with urease treatment. The slightly higher error of measurement in use of immobilized urease is likely associated with the handling of small volumes used in these experiments. For study of urease kinetics, the hydrolysis of urea in synthetic urine was performed at different initial concentrations spanning 4–10 mM. For the same study in real human urine, urea estimation kit was used to assess the amount of urea in various samples of real human urine contributed by different volunteers. Based on the amount of urea estimated across these samples, suitable dilutions of the human urine samples afforded the concentrations of urea spanning 4–10 mM. The estimated concentrations of urea in the different samples of human urine cannot be disclosed here due to exemption from review granted by Institute Ethics Committee of Indian Institute of Technology Gandhinagar. Initial velocity of reactions corresponding to the different substrate concentrations were used to create double-reciprocal (Lineweaver–Burk) plots. The reactions were performed in triplicate and the calculated initial velocities were found to be within 1% of one another in case of synthetic urine and within 5% of one another in case of real human urine. The mean initial velocities measured towards synthetic urine and real human urine were used for the final calculations. The Michaelis–Menten constant (*k*_m_) and the maximum rate (*v*_max_) values of urease nanoconstruct in SUr and HUr were calculated from the Lineweaver–Burk plots.

#### 2.5.2 Effect of pH

The effect of pH of medium on activity of Urease/GA/CS/MIONPs in synthetic and real human urine was investigated by exposing 50 μL of urease nanoconstruct in 2 mL of synthetic and fresh real urine (1/20-fold dilution), respectively, at 25 °C. The Berthelot reaction was used for ammonia detection *via* measurement of absorption of indophenol as described in the previous section. The effect of pH on the activity of urease nanoconstruct was studied at different phosphate buffer saline (PBS) solution in the range of pH 3–10.

#### 2.5.3 Effect of temperature

The effect of temperature of medium on activity of Urease/GA/CS/MIONPs in synthetic and real human urine was investigated by first incubating suspension of urease nanoconstruct (50 mL) at 5 °C intervals in the temperature range of 20–50 °C. 2 mL of real or synthetic urine at 1/20-fold dilution, was added to the suspension at each temperature and ammonia formation was assessed by the Berthelot reaction.

#### 2.5.4 Reusability of urease-immobilized chitosan-coated MIONPs

The reusability of Urease/GA/CS/MIONPs was assessed by separating the constructs by use of neodymium magnet after each batch of reaction with 2 mL of synthetic urine. The immobilized urease was washed twice with buffer followed by incubation with a fresh volume of synthetic urine. The total volume of each batch of reaction was adjusted to a maximum of 2.1 mL. 25 μL of the reactions were used for Berthelot reaction, as described earlier, and comparison of the ammonia formation in each case.

## 3. Results and discussion

### 3.1. Characterization of urease-immobilized chitosan-coated magnetic iron oxide nanoparticles

**Fig. 2:**
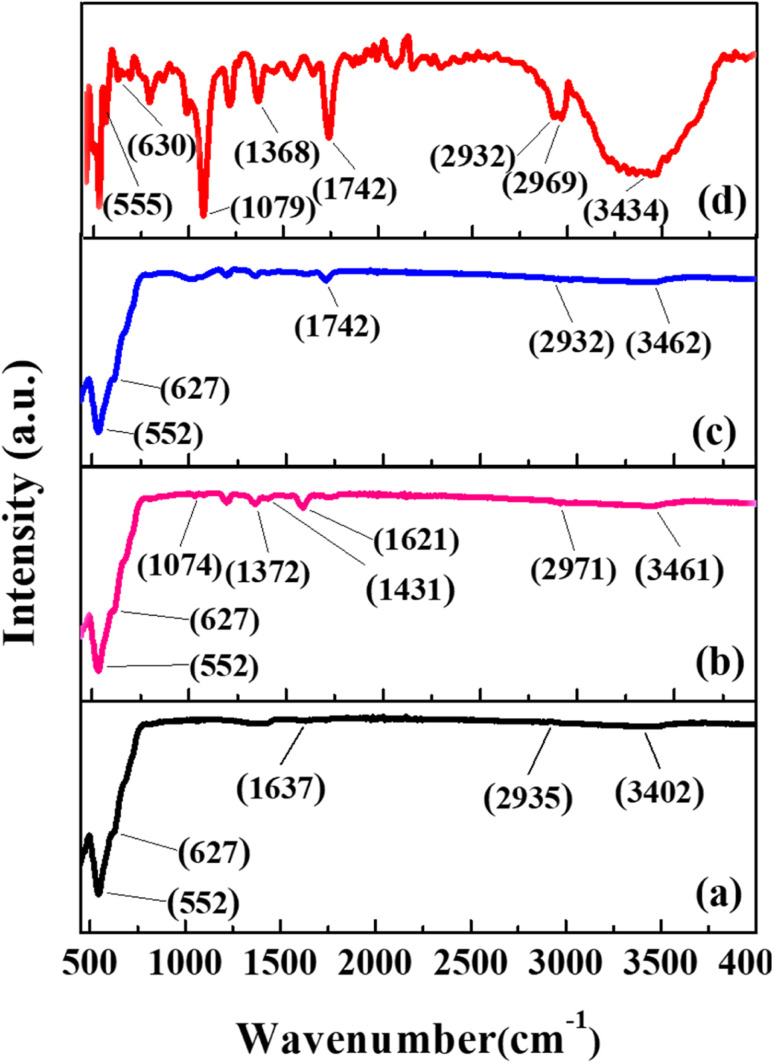
ATR-FTIR spectra of (a) MIONPs, (b) CS/MNIOPs, (c) GA/CS/MIONPs and (d) Urease/GA/CS/MIONPs.

The ATR-FTIR spectra of MIONPs, CS/MIONPs, GA/CS/MIONPs and Urease/GA/CS/MIONPs were measured to confirm the various constructs. For pristine MIONPs, the bands observed at 552 cm^−1^ and 627 cm^−1^ are attributed to the Fe–O stretching vibrations.^37^ The bands appearing at 1637 cm^−1^ and 3402 cm^−1^ arise from –OH bending and –OH stretching, respectively.^38^ Chitosan coating of MIONPs is established by observation of peak at 2971 cm^−1^, corresponding to the –CH– stretching vibrations in chitosan. The amino groups of chitosan are represented by bands at 1621 cm^−1^ and 1431 cm^−1^, from the N–H vibration and C–N vibration, respectively. Additional confirmation of chitosan is obtained from the peak at 1074 cm^−1^, attributed to the ether C–O bond stretching.^32,39^Fig. 2, spectra c depicts ATR-FTIR of GA/CS/MIONPs. The crosslinking of glutaraldehyde with chitosan coated MIONPs is established by the band at 1635 cm^−1^ attributable to imine bond (N=C) stretching.^40^ The crosslinking is further confirmed by band at 1724 cm^−1^ ascribed to the C=O stretching of glutaraldehyde.^41^ The spectrum of urease immobilized on GA/CS/MIONPs (Fig. 2, spectra d) indicates alteration in the broadness of OH/NH_2_ absorption bands at 2900–3500 cm^−1^. The peak at 1368 cm^−1^ is attributed to the amide band of urease.^42^ While the band at 1079 cm^−1^ is due to the stretching vibration of the glycosidic C–O linkage.^43^

**Fig. 3:**
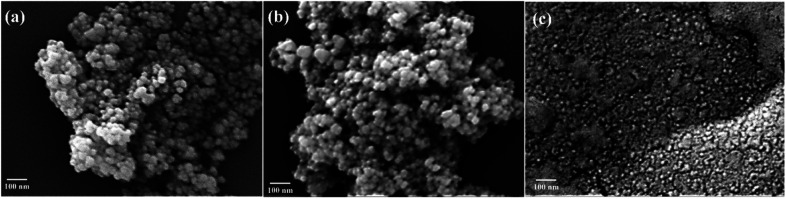
SEM images of (a) MIONPs, (b) GA/CS/MIONPs and (c) Urease/GA/CS/MIONPs.

We performed FE-SEM measurements to investigate changes in the morphology of MIONPs after modification with chitosan and upon immobilization of urease. Fig. 3a, shows spherically shaped MIONPs of particle diameter ∼20 nm. The MIONPs were also characterized by TEM (ESI, Fig. S1†) and revealed nanoparticles in the size range of 15–30 nm. The SEM image of glutaraldehyde crosslinked chitosan modified MIONPs (Fig. 3b), displays a uniform spherical morphology with crystalline structure. SEM of urease immobilized on glutaraldehyde-crosslinked chitosan coated on MIONPs is shown in Fig. 3c and indicate uniform architecture of the nanoconstructs with some reduction in the overall particle size.

**Fig. 4:**
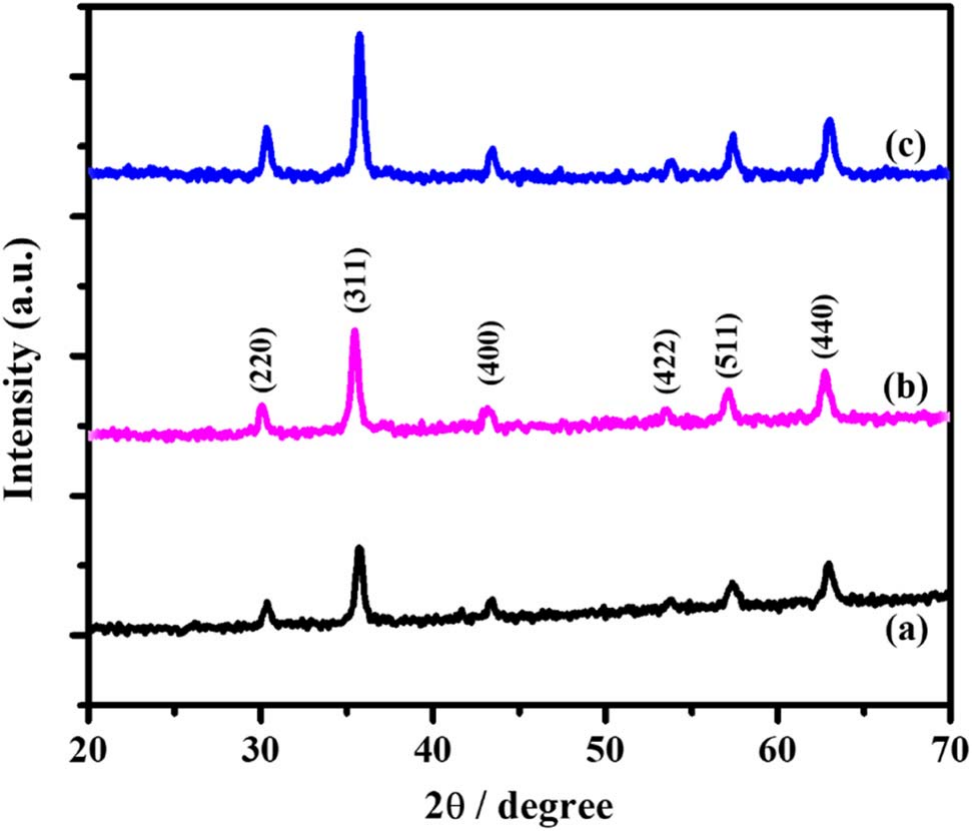
X-ray diffraction patterns of (a) synthesized MIONPs nanoparticles, (b) chitosan coated iron oxide nanoparticles (CS/MIONPS) and (c) glutaraldehyde crosslinked GA/CS/MIONPs.

The structural properties of magnetic iron oxide particles (MIONPs) and the glutaraldehyde crosslinked chitosan modified magnetic iron oxide nanoparticles (GA/CS/MIONPs) were explored by X-ray diffraction (XRD). The XRD patterns of the glutaraldehyde crosslinked chitosan modified magnetic iron oxide nanoparticles (GA/CS/MIONPs), pristine iron oxide nanoparticles (MIONPs) and chitosan coated iron oxide nanoparticles (CS/MIONPs) are shown in Fig. 4. The diffraction peaks of pristine iron oxide nanoparticles, chitosan coated MIONPS and the glutaraldehyde crosslinked CS/MIONPs were matched with the standard cubic Fe_3_O_4_ (magnetite), 30.28, 35.657, 43.39, 53.7, 57.31 and 62.91 (2*θ*) which refers to the (220), (311), (400), (422), (511) and (440) planes.^44^ The characteristic peaks at 2*θ*, 21.228 and 33.158 were absent in the diffraction pattern suggesting the absence of other oxides namely goethite (FeOOH) and hematite (Fe_2_O_3_).^38^ These results confirm that the synthesized iron oxide nanoparticles, namely the chitosan coated MIONPS and the glutaraldehyde crosslinked CS/MIONPs possess a magnetite phase, *i.e.*, Fe_3_O_4_ crystal structure with no impurities.

The Debye–Scherrer formula is used to determine the average particle size of Fe_3_O_4_ nanoparticles.^45^1
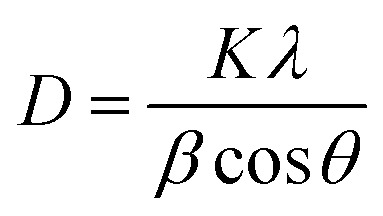
where *D* is the average size of the crystallites, *K* is the shape factor (*K* = 0.94, considering spherical magnetite nanoparticles), *λ* is the X-ray wavelength and *β* is the full width at half-maximum of the highest intensity reflection at diffraction angle *θ*. The estimated average size of Fe_3_O_4_ was approximately 20 nm in each of pristine MIONPs nanoparticles, chitosan coated iron oxide nanoparticles (CS/MIONPs) and GA/CS/MIONPs. In this context, it should be noted that the size of the synthesized MIONPs has also been confirmed from transmission electron microscopy (TEM) as shown in Fig. S1, ESI.† These results are consistent with our scanning electron microscopy (SEM) analysis. The average hydrodynamic size of MIONPs, CS/MIONPs and GA/CS/MIONPs was also investigated by dynamic light scattering (see ESI, Table S1†). The EDX spectra of the MIONPs, GA/CS/MIONPs and Urease/GA/CS/MIONPs provides additional support for the immobilization of urease enzyme on the GA/CS/MIONPs (see ESI, Fig. S2†).

**Fig. 5:**
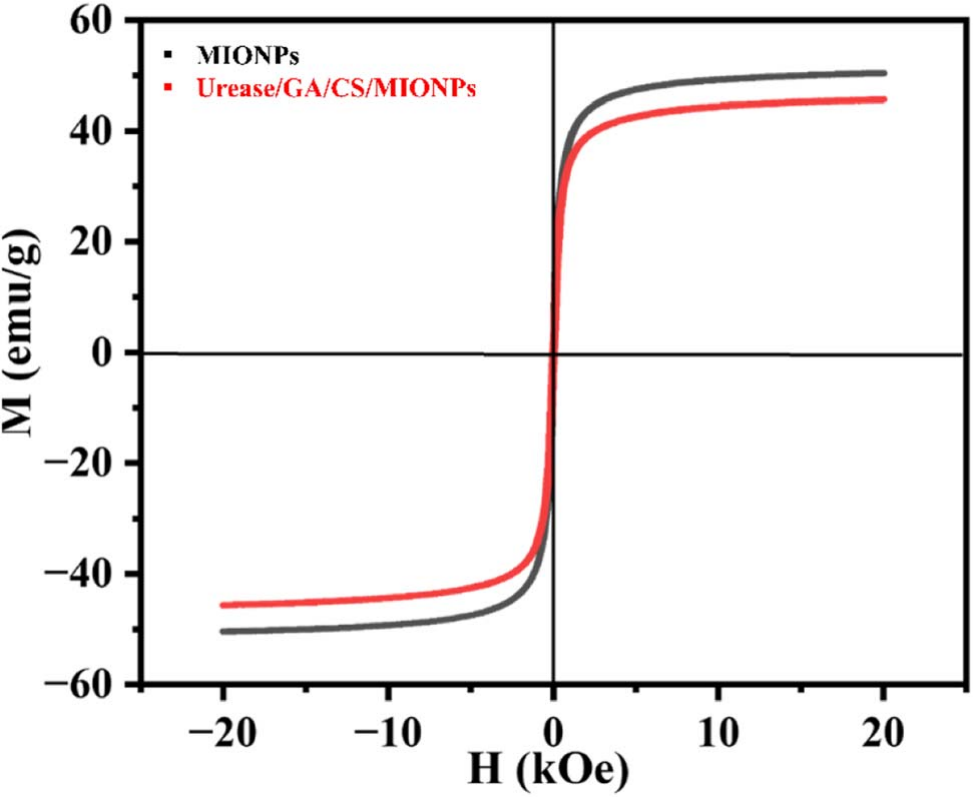
Plot of magnetization (*M*) as a function of applied magnetic field (*H*) for MIONPs nanoparticles and the Urease/GA/CS/MIONPs, at room temperature.

The magnetic properties of magnetic iron oxide particles (MIONPs) and the urease immobilized glutaraldehyde crosslinked chitosan modified magnetic iron oxide nanoparticles (Urease/GA/CS/MIONPs) were analysed using vibrating sample magnetometry (VSM) at room temperature. Fig. 5 shows the plots of magnetization *versus* the applied magnetic field strength of Urease/GA/CS/MIONPs and pristine MIONPs. The results indicate that both pristine MIONPs and Urease/GA/CS/MIONPs exhibit superparamagnetic behaviour with negligible coercivity and remanence.^46^ Superparamagnetism, characterized by the ability of a material to become magnetized when subjected to a magnetic field but lacking permanent magnetization (remanence) upon field removal, is crucial for applications in magnetic separation.^47^ In the range of an applied field between 6.2 to 20 kOe the induced magnetization in the urease nanoconstruct Urease/GA/CS/MIONPs was measured to be around 43–45.5 emu g^−1^ at room temperature. On the other hand, pristine MIONPs showed an induced magnetization ranging from 48–50.4 emu g^−1^ at this range (H ∼6.2, to 20 kOe) and room temperature, which aligns with literature reports.^48,49^ The VSM results show that the saturation magnetization of Urease/GA/CS/MIONPs is lower, than that of MIONPs. This is possibly because the MIONPs are incorporated and dispersed within the nanoconstruct. The magnetic properties of Urease/GA/CS/MIONPs enable their straight-forward separation from the aqueous solution using a magnetic field.

### 3.2. Effect of urine dilution on urease-catalyzed hydrolysis

**Fig. 6:**
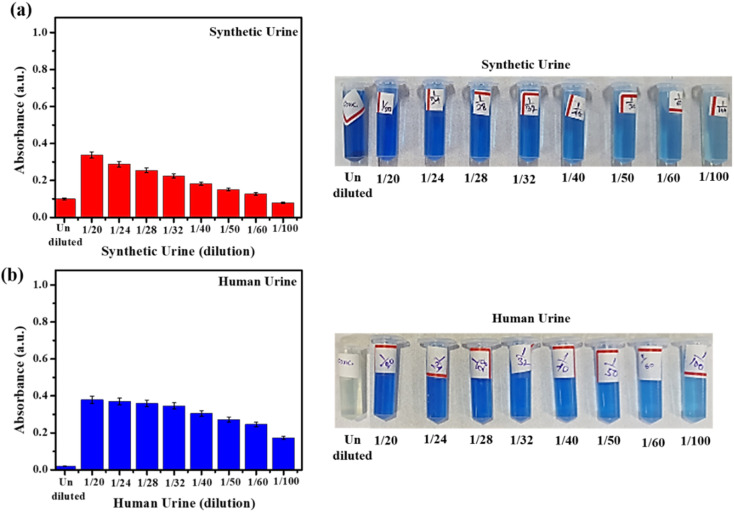
Effect of dilution on the monitoring of ammonium ions at 25 °C, (a) in synthetic urine and (b) in human urine, without the application of urease nanoconstruct treatment. The Berthelot reaction is performed in DI water. The standard error of measurement was in the range of 1–3%.

**Fig. 7:**
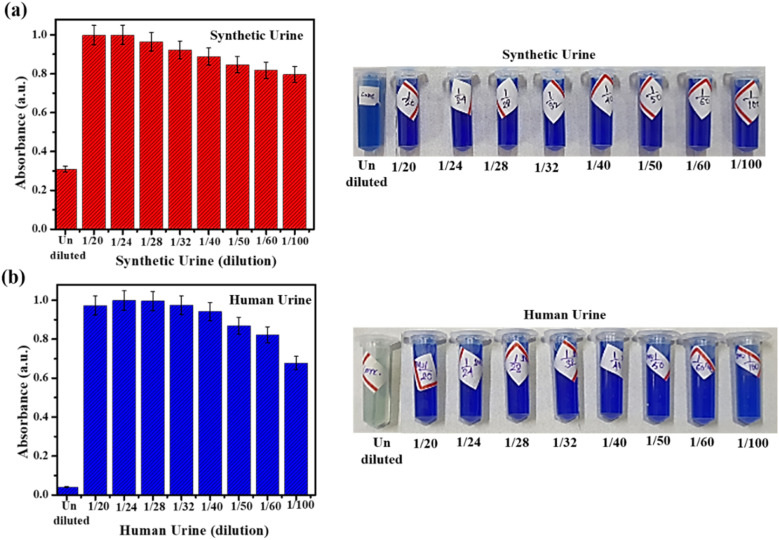
Effect of dilution on urea hydrolysis and ammonium ion monitoring following treatment with urease nanoconstruct at 25 °C, (a) in synthetic urine (SUr), and (b) in real fresh human urine (HUr). The Berthelot reaction is conducted in deionized water. The Berthelot reaction is performed in DI water. The standard error of measurement was in the range of 1–8%.

We began our investigations into immobilized urease activity by first assessing the effect of urine dilution on the enzyme-catalyzed hydrolysis. The prepared urease nanoconstruct was separately exposed to synthetic (SUr) and real human urine (HUr) at different dilutions, followed by ammonia measurement *via* UV-visible spectrophotometry. The formation of indophenol *via* Berthelot reaction, is linearly correlated to ammonia concentration. Urease treatment of urine followed by Berthelot reaction is used for clinical assessment of urea levels. These methods require significant dilution (∼50-fold) of urine primarily due to modest sensitivity and limit of quantification of the Berthelot reaction. Nevertheless, the systematic comparison of undiluted SUr and HUr with respect to ammonia content measured by Berthelot method are not widely reported. Thus, we first investigated the initial ammonium ion levels in SUr and HUr, without any enzyme-catalyzed hydrolysis reaction being performed. As shown in Fig. 6a, dilution of SUr results in a concomitant decrease in indophenol absorbance confirming the expected decrease of ammonia concentration. Interestingly, while the undiluted HUr failed to register indophenol formation, dilution by 1/20-fold resulted in highest indophenol absorbance corresponding to its formation (Fig. 6b). Subsequent dilutions of HUr produce a gradual decrease in absorbance. Dilutions of both SUr and HUr that are less than 1/20-fold display progressively lower levels of indophenol formation till the absorbance corresponding to the undiluted state (data not shown). Progressive dilution of both SUr and HUr is expected to generate decreasing levels of ions. The undiluted SUr with or without treatment with immobilized urease registers indophenol formation (Fig. 6a and 7a). The unusual behaviour of undiluted HUr with respect to lack of indophenol formation is possibly due to higher content of organic substances in the HUr creating interference with Berthelot reaction. The application of Urease/GA/CS/MIONPs on SUr and HUr at various dilutions is shown in Fig. 7. Progressive dilution of both SUr and HUr is expected to generate decreasing levels of all ions including the NH_4_^+^ arising from urea hydrolysis. This is confirmed by conductance measurements on the immobilized urease-treated SUr and HUr samples (see ESI, Fig. S3†). Notably, the conductance measurements are not specific to NH_4_^+^ but capture the overall decrease in ionic strength with dilution. Taken together, these results suggest that immobilized urease activity on the undiluted SUr and HUr is suppressed and high levels of activity can be observed for high-fold dilution on both SUr and HUr. Enzyme urease is known to be inhibited by several mono-, di- and multi-valent metal ions.^50,51^ While the presence of such metal ions in the SUr and HUr used in our experiments is unlikely, we were intrigued by the suppression of immobilized urease activity in undiluted SUr. To probe this observation further, we treated samples containing identical amount of urea and ammonium chloride as SUr (see ESI, Table S2†), but only containing variable concentrations of KCl (50 mM, 100 mM, and 200 mM), and lacking all other components of SUr. While these simplified samples did not contain the diversity of ions present in SUr, we observed progressive suppression of ammonium formation in presence of increasing concentrations of KCl (ESI, Fig. S4†). This result suggests the possible inaccessibility of immobilized urease in presence of high salt concentrations, that may be arising due to heightened collapse of the enzyme on the nanoparticle surface. In the context of urine, the reaction catalyzed by urease relies on the ease of availability of urea.^19^

2 NH_2_(CO)NH_2_ → 2NH_4_^+^ + HCO_3_^−^ + OH^−^

A few aspects emerge from comparison of the performance of immobilized urease on SUr and HUr. First, the total ammonium content in urease-treated diluted SUr and HUr is several-fold higher compared to the untreated SUr and HUr (Fig. 6 and 7). Notwithstanding the disruption in Berthelot reaction in concentrated HUr, this suggests that the designed activity of immobilized urease towards hydrolysis of resident urea to ammonia is successful. In fact, a measurable increase in NH_4_^+^ is observed comparing the treated *versus* untreated samples of HUr (Fig. 6b and 7b). Second, the NH_4_^+^ produced by urease treatment of SUr and HUr is comparable across the range of dilutions studied. The standard error of measurements of the NH_4_^+^ produced for Urease/GA/CS/MIONPs reactions with SUr and HUr was in the range of 1–8% and were likely due to the reactions being performed in small volumes along with constraints in sensitivity of the measurements. The observed interplay of diluted urine vis-à-vis performance of immobilized urease has received scant attention in the past, and is likely to be of interest to researchers working on urine treatment. The optimum NH_4_^+^ production in specific dilutions of SUr and HUr supports the practical relevance of the Urease/GA/CS/MIONPs in a real urine treatment setup. For example, source-separated urinals could be easily adapted to generate suitably diluted urine for immediate treatment by the immobilized urease constructs. The comparable performance of Urease/GA/CS/MIONPs towards HUr and SUr is noteworthy considering the plethora of components in HUr that are largely unrepresented in SUr. This part of the study fulfils one of our objectives of the current work considering absence of reports comparing the behaviour of immobilized urease between SUr and HUr. In particular, except for creatinine, SUr does not contain any organic compounds at all in contrast to the typical clinical profile of HUr.^52^ Apart from indicating the robust behaviour of the immobilized urease constructs, these results also imply that SUr can be considered as a viable reaction medium for further experimentation with and refinement of the immobilized urease strategy.

We calculated the ammonium concentration corresponding to 1/20 and 1/24-fold diluted SUr and HUr upon treatment with immobilized urease (ESI, Table S3†). The higher concentration of NH_4_^+^ released in Urease/GA/CS/MIONPs treated *versus* untreated urine proves the rationale for deploying the immobilized urease construct. The concentration of NH_4_^+^ for the urease-catalyzed hydrolysis of SUr and HUr are comparable across the optimal-folds of dilution, and reaffirm SUr as a robust medium for replicating effects observed in human urine.

### 3.3. pH and temperature effects and reusability of immobilized urease

**Fig. 8:**
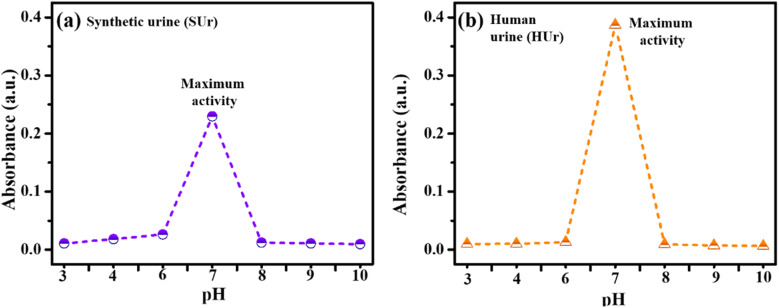
Effect of pH on urea hydrolysis and ammonium ion monitoring, (a) in synthetic urine (SUr), and (b) in real fresh human urine (HUr), following treatment with urease nanoconstruct. Both SUr and HUr are diluted at a ratio of 1/20 in PBS solution, with pH ranging from 3 to 10 at a temperature of 25 °C. The Berthelot reaction is performed in the PBS buffer solution.

The optimum pH for the immobilized urease on GA/CS/MIONPs was determined by conducting the immobilized urease catalyzed hydrolysis of suitably diluted SUr and HUr (1/20 dilution of SUr and HUr) across a phosphate buffer saline (PBS) solution (*i.e.*, pH range of 3-10) at room temperature. While these experiments were performed in PBS and phosphate at pH 7 has been reported to suppress urease activity^53^ we were able to observe sufficient difference in enzyme activity across the pH range studies. The absorbance value is low as compare to the value obtained in the dilution study (Fig. 6 and 7) because the urease-catalyzed hydrolysis of urea has been found to be competitively inhibited by phosphate at pH 7.0^53^. As shown in Fig. 8a and b, the pH effect on the enzyme activity revealed that the optimal pH is around 7 in both SUr and HUr solutions. Immobilization of enzymes on solid supports is fraught with changes in structural attributes of the catalysts which could unfavourably alter their performance depending on the pH of the medium. The optimum pH of 7.0 for our GA/CS/MIONPs bodes well for their use towards transforming urine at a bulk scale precluding the need for substantial pH buffering of the reaction. Our results are comparable to previous reports on immobilized urease activity.^54–56^

**Fig. 9:**
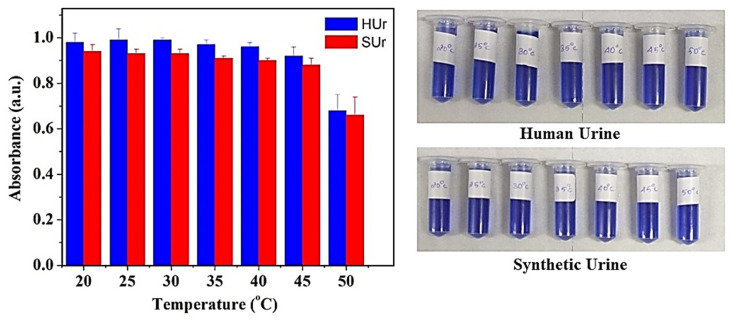
Effect of temperature on urea hydrolysis and ammonium ion monitoring in both human urine (HUr) and synthetic urine (SUr) following treatment with urease nanoconstruct. Both SUr and HUr are diluted at a ratio of 1/20. The Berthelot reaction is carried out in DI water. The standard error of measurement was in the range of 1–10%.

**Fig. 10:**
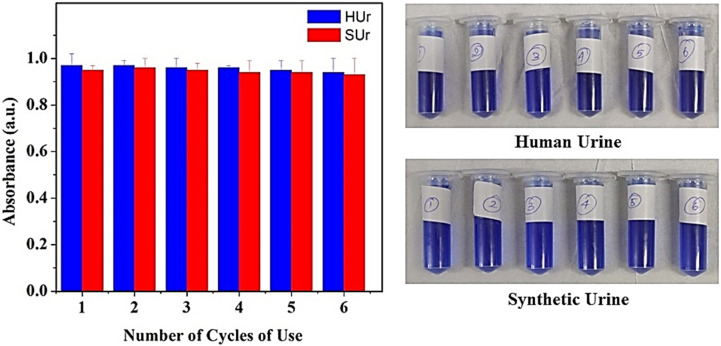
Evaluation of urease nanoconstruct and ammonium ion monitoring over six cycles of application in both human urine (HUr) and synthetic urine (SUr). Both SUr and HUr are diluted at a ratio of 1/20. The Berthelot reaction is performed in DI water. The standard error of measurement was in the range of 1–10%.

Similarly, a study of the performance of our immobilized urease across a range of temperatures revealed consistent urea-hydrolyzing action across 20–45 °C with a drop-in activity only beyond 50 °C (Fig. 9). The robust behaviour of the immobilized urease over a broad temperature range underscores its potential for field deployment. In this regard, the immobilization of urease on magnetic iron oxide nanoparticles had been sought to achieve reusability of enzyme. We tested single batches of immobilized urease across six cycles of treatment on HUr and SUr. The number of cycles of reuse of immobilized urease was based on the reaction volume being used. As shown in Fig. 10, negligible loss of enzyme activity was observed over the six cycles tested. The standard error of measurements of NH_4_^+^ produced by Urease/GA/CS/MIONPs as a function of temperature was in the range of 1–10%, with the higher temperatures possibly resulting in some enzyme denaturation. Use of larger reaction volumes is likely to facilitate accurate measurements on the reuse of immobilized urease over a significantly larger number of cycles (data not shown).

### 3.4. Kinetics of immobilized urease towards SUr and HUr

**Fig. 11:**
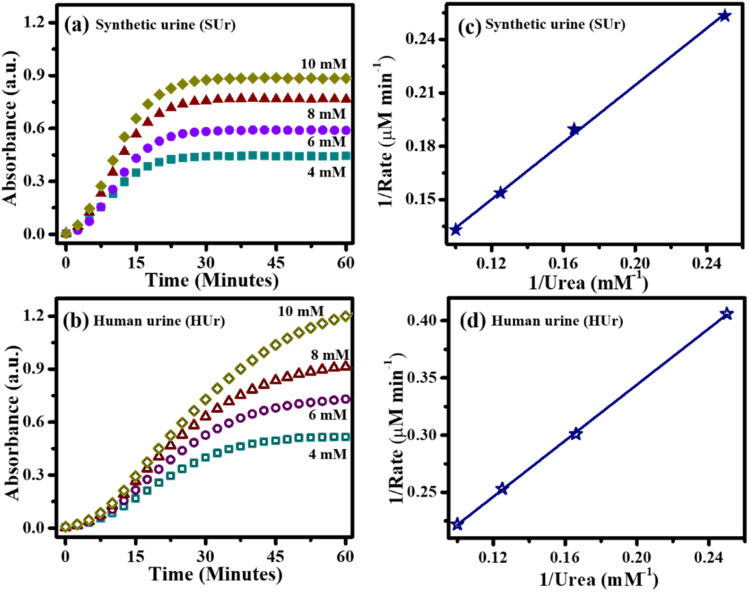
Urease–urea reaction kinetic plots (a), (b) at different time intervals for several concentrations of urea, (c) and (d) Lineweaver–Burk plot of urease immobilized GA/CS/MIONPs in synthetic urine (SUr) and real fresh human urine (HUr).

We next investigated the kinetics of urease nanoconstructs towards SUr and HUr. Based on the Lineweaver–Burk formalism of Michaelis–Menten kinetics eqn (3),^54^ the kinetic parameters of the urease-catalyzed hydrolysis of urea for both synthetic and real fresh urine could be determined.^55^3
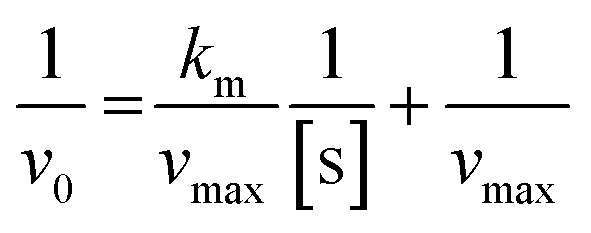
here, *v*_0_ is the rate of urea hydrolysis in urine, [s] is the concentration of urea in urine, *v*_max_, the maximum rate of reaction, and *k*_m_ is the Michaelis constant (mM). The change of the reaction rate of urease, when exposed to different concentrations of urea contained in SUr and HUr, can be used to determine the standard kinetic parameters of maximum rate (*v*_max_) and Michaelis constant (*k*_m_). For this study, urease nanoconstruct performance was studied at concentrations of urea spanning 4–10 mM in both SUr and HUr. The *k*_m_ value is a measure of the affinity between enzyme and substrate. A lower enzyme–substrate affinity is indicated by a higher *k*_m_ value and *vice versa*. The *v*_max_ value is indicative of the enzyme's theoretical maximal rate.^54,57^ The maximum velocity of urease can be estimated from the intercept of the straight line (1/*v*_max_), while the slope provides the ratio of the Michaelis constant to the maximum hydrolysis rate (*k*_m_/*v*_max_). The Lineweaver–Burk method results in comparable *k*_m_ of urease immobilized GA/CS/MIONPs against SUr (14.86 mM) and HUr (12.36 mM) (Fig. 11). The *v*_max_ for urease-immobilized GA/CS/MIONPs was 18.55 μM min^−1^ and 10.10 μM min^−1^, in SUr and HUr, respectively.

Our calculated kinetic parameters are consistent with those reported previously for various immobilized urease constructs (see ESI, Table S4†). Immobilization of urease across different solid supports has been shown to increase *k*_m_ along with a decrease in *v*_max_.^57^ These observations are justified based on enzyme immobilization restricting free movement of the enzyme or the diffusion limitation that may delay the urea–urease interaction following the fixation of the enzyme on a solid support. Alternatively, conformational disruptions that frequently follow the covalent bonding of the enzyme onto the solid support *via* multi-point attachment may also lower the efficacy of the enzyme.^57^ Comparing the kinetic parameters of urease immobilized GA/CS/MIONPs, it is evident that access to substrate (urea) is not significantly affected in HUr compared to SUr. In contrast, the *v*_max_ for HUr of 10.10 μM min^−1^ is significantly lowered to 18.55 μM min^−1^ in SUr. Such a sizeable drop in *v*_max_ with only modest change in *k*_m_ can be likened to an uncompetitive state of inhibition of the urease in HUr. A comparison of the kinetic parameters of our Urease/GA/CS/MIONPs between SUr and HUr is notable considering the near-absence of such comparisons in published reports on immobilized urease. HUr is considerably more complex with a diverse set of organic and biomolecular constituents, that are absent in SUr. The kinetic parameters for Urease/GA/CS/MIONPs for HUr and SUr are comparable to the kinetics of several other immobilized urease constructs (Table S4†). The kinetic profile of Urease/GA/CS/MIONPs in combination with its robustness and ease-of-use, preparation, and standardization, describe an attractive urea-hydrolysing agent. The classical portrayal of uncompetitive inhibition involves the depletion of enzyme–substrate complex due to the presence of a specific entity that can thus be termed an inhibitor. Different forms of the enzyme substrate for urea, such as methylurea,^58^ hydroxyureas,^59^ thioureas, and selenoureas,^60^ served as inhibitors of urease. A recently patented compound, known as 17, which is based on urea, exhibited notable activity against urease with an IC50 value of 1.25 μM.^61^ Furthermore, within the pH range of 5.0–8.0, phosphate acts as a competitive inhibitor of urease that is dependent on pH. However, its inhibitory effect becomes insignificant at pH levels above 7.5–8.0.^62,63^ Considering the complexity in composition of HUr it would be naïve to speculate on any one inhibitory agent. Instead, HUr may be better interpreted as a medium that interferes with the catalytic steps of urease. Overall, the urease immobilized GA/CS/MIONPs exert comparable hydrolysis of urea in SUr and HUr and suggest the suitability of SUr as a synthetic mimic of human urine while also highlighting the robust character of the nanoconstruct.

## 4. Conclusion

Nutrient generation and its recovery from human liquid waste can aid agricultural practices by providing sustainable fertilizers. Human urine is a nutrient-rich material that deserves to be researched for accessing the nitrogen content. While synthetic urine has been extensively used for research on urine-related molecular processes, a systematic comparison of urease-facilitated hydrolysis of synthetic *versus* real human urine has not been reported. Also, the effect of urine dilution on immobilized urease activity is sparsely reported in general, and even less so for a comparison of synthetic *versus* real human urine. In this work, we have synthesized and examined the ability of urease immobilized magnetic iron oxide nanoparticles (Urease/GA/CS/MIONPs) to hydrolyse urea present in synthetic and real fresh human urine. The Urease/GA/CS/MIONPs reported in this work display comparable hydrolysis of urea in SUr and HUr. 1/20 fold dilution of SUr and 1/24 fold dilution of HUr result in optimum urea hydrolysis by the Urease/GA/CS/MIONPs. The immobilized urease constructs function effectively across a broad range of ambient temperatures covering 20–45 °C, and can be reused across multiple batches of reaction with urine. The Urease/GA/CS/MIONPs display superparamagnetic behaviour that facilitates straight-forward separation from the reaction medium. The kinetic analysis indicates the *k*_m_ value for nanoparticle immobilized urease as 14.86 mM towards SUr and 12.36 mM for HUr, and the corresponding *v*_max_ of 18.55 μM min^−1^ and 10.10 μM min^−1^, in SUr and HUr, respectively. The kinetic parameters of Urease/GA/CS/MIONPs compare effectively with other immobilized urease constructs. The ease of preparation, characterization, and standardization of these constructs, compared to previously reported immobilized ureases, makes them an attractive candidate for use in urine treatment. The robust performance of Urease/GA/CS/MIONPs is encouraging for urine treatment under ambient conditions facilitating subsequent harvesting of the ammonia.^64^ In particular, the deployment of immobilized urease coupled with adsorptive extraction of the released NH_4_^+^ suggests is an attractive approach towards human liquid waste management and is currently being investigated in our laboratory.

## Statement of informed consent

Informed consent was obtained from all volunteers who contributed human urine to this work.

## Conflicts of interest

There are no conflicts of interest to declare.

## Supplementary Material

RA-014-D3RA07606G-s001

## References

[cit1] Galloway J. N., Aber J. D., Erisman J. W., Seitzinger S. P., Howarth R. W., Cowling E. B., Cosby B. J. (2003). The nitrogen cascade. Bioscience.

[cit2] Yapicioglu A., Dincer I. (2019). A review on clean ammonia as a potential fuel for power generators. Renewable Sustainable Energy Rev..

[cit3] Baker M. , in Overview of Industrial Urea Markets: Application and Opportunities, TFI Fertilizer Technology and Outlook Conference, Philadelphia, PA, 2012

[cit4] Patel A., Mungray A. A., Mungray A. K. (2020). Technologies for the recovery of nutrients, water and energy from human urine: A review. Chemosphere.

[cit5] Moya B., Parker A., Sakrabani R. (2019). Challenges to the use of fertilisers derived from human excreta: The case of vegetable exports from Kenya to Europe and influence of certification systems. Food Policy.

[cit6] Metcalf Eddy , Abu-Orf M., Bowden G., Burton F. L., Pfrang W., Stensel H. D., Tchobanoglous G., Tsuchihashi R. and AECOM, Wastewater Engineering: Treatment and Resource Recovery, McGraw Hill Education, 2014

[cit7] Ray H., Perreault F., Boyer T. H. (2020). Rejection of nitrogen species in real fresh and hydrolyzed human urine by reverse osmosis and nanofiltration. J. Environ. Chem. Eng..

[cit8] Pradhan S. K., Mikola A., Vahala R. (2017). Nitrogen and phosphorus harvesting from human urine using a stripping, absorption, and precipitation process. Environ. Sci. Technol..

[cit9] Krajewska B., Ureases I. (2009). Functional, catalytic and kinetic properties: A review. J. Mol. Catal. B: Enzym..

[cit10] Mobley H., Hausinger R. (1989). Microbial ureases: significance, regulation, and molecular characterization. Microbiol. Rev..

[cit11] Udert K. M., Larsen T. A., Biebow M., Gujer W. (2003). Urea hydrolysis and precipitation dynamics in a urine-collecting system. Water Res..

[cit12] Ukwuani A. T., Tao W. (2016). Developing a vacuum thermal stripping – acid absorption process for ammonia recovery from anaerobic digester effluent. Water Res..

[cit13] Kim J. K., Park K. J., Cho K. S., Nam S.-W., Park T.-J., Bajpai R. (2005). Aerobic nitrification–denitrification by heterotrophic Bacillus strains. Bioresour. Technol..

[cit14] Rose C., Parker A., Jefferson B., Cartmell E. (2015). The characterization of feces and urine: a review of the literature to inform advanced treatment technology. Crit. Rev. Environ. Sci. Technol..

[cit15] Lind B.-B., Ban Z., Bydén S. (2000). Nutrient recovery from human urine by struvite crystallization with ammonia adsorption on zeolite and wollastonite. Bioresour. Technol..

[cit16] Höglund C., Stenström T., Jönsson H., Sundin A. (1998). Evaluation of faecal contamination and microbial die-off in urine separating sewage systems. Water Sci. Technol..

[cit17] Hanaerus A., Hellström D., Johansson E. (1996). Conversion of urea during storage of humane urine. Vatten.

[cit18] Deng H.-H., Hong G.-L., Lin F.-L., Liu A.-L., Xia X.-H., Chen W. (2016). Colorimetric detection of urea, urease, and urease inhibitor based on the peroxidase-like activity of gold nanoparticles. Anal. Chim. Acta.

[cit19] Ray H., Saetta D., Boyer T. H. (2018). Characterization of urea hydrolysis in fresh human urine and inhibition by chemical addition. Environ. Sci.: Water Res. Technol..

[cit20] Murata H., Cummings C. S., Koepsel R. R., Russell A. J. (2013). Polymer-based protein engineering can rationally tune enzyme activity, pH-dependence, and stability. Biomacromolecules.

[cit21] Fujita K., MacFarlane D. R., Forsyth M., Yoshizawa-Fujita M., Murata K., Nakamura N., Ohno H. (2007). Solubility and stability of cytochrome c in hydrated ionic liquids: effect of oxo acid residues and kosmotropicity. Biomacromolecules.

[cit22] Yasutaka K., Takato Y., Takashi K., Kohsuke M., Hiromi Y. (2011). Enhancement in adsorption and catalytic activity of enzymes immobilized on phosphorus-and calcium-modified MCM-41. J. Phys. Chem. B.

[cit23] De Hoog H.-P. M., Arends I. W., Rowan A. E., Cornelissen J. J., Nolte R. J. (2010). A hydrogel-based enzyme-loaded polymersome reactor. Nanoscale.

[cit24] Kato K., Nishida M., Ito K., Tomita M. (2012). Characterization of silica particles prepared via urease-catalyzed urea hydrolysis and activity of urease in sol–gel silica matrix. Appl. Surf. Sci..

[cit25] Silva G. S., Oliveira P. C., Giordani D. S., Castro H. F. d. (2011). Chitosan/siloxane hybrid polymer: synthesis, characterization and performance as a support for immobilizing enzyme. J. Braz. Chem. Soc..

[cit26] Kirdeciler S. K., Soy E., Öztürk S., Kucherenko I., Soldatkin O., Dzyadevych S., Akata B. (2011). A novel urea conductometric biosensor based on zeolite immobilized urease. Talanta.

[cit27] Katz E. and Pita M., Biomedical applications of magnetic particles. Fine Particles in Medicine and Pharmacy, 2012, pp. 147–173

[cit28] Zhu Y., Kaskel S., Shi J., Wage T., van Pée K.-H. (2007). Immobilization of Trametes versicolor laccase on magnetically separable mesoporous silica spheres. Chem. Mater..

[cit29] Borole A. , Dai S., Cheng C. L., Rodriguez M. and Davison B. H., in Performance of chloroperoxidase stabilization in mesoporous sol-gel glass using in situ glucose oxidase peroxide generation, Proceedings of the Twenty-Fifth Symposium on Biotechnology for Fuels and Chemicals Held May 4–7, 2003, in Breckenridge, CO, 2004, Springer, 2004, pp. 273–285 10.1385/abab:113:1-3:273 15054212

[cit30] Kumar S., Morya V., Gadhavi J., Vishnoi A., Singh J., Datta B. (2019). Investigation of nanoparticle immobilized cellulase: nanoparticle identity, linker length and polyphenol hydrolysis. Heliyon.

[cit31] Singh R., Datta B. (2022). Banana Peel Powder as an Effective Multilayer Adsorbent of Ammonium Ions. Ind. Eng. Chem. Res..

[cit32] Pham X. N., Nguyen T. P., Pham T. N., Tran T. T. N., Tran T. V. T. (2016). Synthesis and characterization of chitosan-coated magnetite nanoparticles and their application in curcumin drug delivery. Adv. Nat. Sci.: Nanosci. Nanotechnol..

[cit33] Kumar S., Sharma P., Ratrey P., Datta B. (2016). Reusable nanobiocatalysts for the efficient extraction of pigments from orange peel. J. Food Sci. Technol..

[cit34] Yang D., Qiu L., Yang Y. (2016). Efficient Adsorption of Methyl Orange Using a Modified Chitosan Magnetic Composite Adsorbent. J. Chem. Eng. Data.

[cit35] Krom M. D. (1980). Spectrophotometric determination of ammonia: a study of a modified Berthelot reaction using salicylate and dichloroisocyanurate. Analyst.

[cit36] Kutcherlapati S. R., Yeole N., Jana T. (2016). Urease immobilized polymer hydrogel: Long-term stability and enhancement of enzymatic activity. J. Colloid Interface Sci..

[cit37] Sahoo Y., Goodarzi A., Swihart M. T., Ohulchanskyy T. Y., Kaur N., Furlani E. P., Prasad P. N. (2005). Aqueous ferrofluid of magnetite nanoparticles: fluorescence labeling and magnetophoretic control. J. Phys. Chem. B.

[cit38] Singh R., Pal D., Chattopadhyay S. (2020). Target-specific superparamagnetic hydrogel with excellent pH sensitivity and reversibility: a promising platform for biomedical applications. ACS Omega.

[cit39] Shukla S., Jadaun A., Arora V., Sinha R. K., Biyani N., Jain V. (2015). In vitro toxicity assessment of chitosan oligosaccharide coated iron oxide nanoparticles. Toxicol. Rep..

[cit40] Ingole P. G., Thakare N. R., Kim K., Bajaj H. C., Singh K., Lee H. (2013). Preparation, characterization and performance evaluation of separation of alcohol using crosslinked membrane materials. New J. Chem..

[cit41] Mohapatra B. R. (2021). Characterization of β-mannanase extracted from a novel Streptomyces species Alg-S25 immobilized on chitosan nanoparticles. Biotechnol. Biotechnol. Equip..

[cit42] Tamaddon F., Arab D. (2019). Urease covalently immobilized on cotton-derived nanocellulose-dialdehyde for urea detection and urea-based multicomponent synthesis of tetrahydro-pyrazolopyridines in water. RSC Adv..

[cit43] Almulaiky Y. Q., Al-Harbi S. A. (2022). Preparation of a calcium alginate-coated polypyrrole/silver nanocomposite for site-specific immobilization of polygalacturonase with high reusability and enhanced stability. Catal. Lett..

[cit44] Reddy N. N., Ravindra S., Reddy N. M., Rajinikanth V., Raju K. M., Vallabhapurapu V. S. (2015). Temperature responsive hydrogel magnetic nanocomposites for hyperthermia and metal extraction applications. J. Magn. Magn. Mater..

[cit45] Radoń A., Drygała A., Hawełek Ł., Łukowiec D. (2017). Structure and optical properties of Fe3O4 nanoparticles synthesized by co-precipitation method with different organic modifiers. Mater. Charact..

[cit46] Singh R., Pal D., Chattopadhyay S. (2020). Target-specific superparamagnetic hydrogel with excellent pH sensitivity and reversibility: a promising platform for biomedical applications. ACS Omega.

[cit47] Guo W., Hu W., Pan J., Zhou H., Guan W., Wang X., Xu L. (2011). Selective adsorption and separation of BPA from aqueous solution using novel molecularly imprinted polymers based on kaolinite/Fe3O4 composites. Chem. Eng. J..

[cit48] Huang Y., Liu M., Chen J., Gao C., Gong Q. (2012). A novel magnetic triple-responsive composite semi-IPN hydrogels for targeted and controlled drug delivery. Eur. Polym. J..

[cit49] Mikami R., Taguchi M., Yamada K., Suzuki K., Sato O., Einaga Y. (2004). Reversible Photo-Switching of the Magnetization of Iron Oxide Nanoparticles at Room Temperature. Angew. Chem., Int. Ed..

[cit50] Krajewska B. (2008). Mono-(Ag, Hg) and di-(Cu, Hg) valent metal ions effects on the activity of jack bean urease. Probing the modes of metal binding to the enzyme. J. Enzyme Inhib. Med. Chem..

[cit51] Fopase R., Nayak S., Mohanta M., Kale P., Paramasivan B. (2019). Inhibition assays of free and immobilized urease for detecting hexavalent chromium in water samples. 3 Biotech.

[cit52] Cunarro J. A., Weiner M. W. (1974). A comparison of methods for measuring urinary ammonium. Kidney Int..

[cit53] Harmon K. M., Niemann C. (1949). The competitive inhibition of of the urease-catalyzed hydrolysis of urea by phosphate. J. Biol. Chem..

[cit54] Yang L., Liu X., Zhou N., Tian Y. (2019). Characteristics of refold acid urease immobilized covalently by graphene oxide-chitosan composite beads. J. Biosci. Bioeng..

[cit55] Tamaddon F., Arab D., Ahmadi-AhmadAbadi E. (2020). Urease immobilization on magnetic micro/nano-cellulose dialdehydes: Urease inhibitory of Biginelli product in Hantzsch reaction by urea. Carbohydr. Polym..

[cit56] Srivastava P. K., Kayastha A. M. (2001). Characterization of gelatin-immobilized pigeonpea urease and preparation of a new urea biosensor. Biotechnol. Appl. Biochem..

[cit57] Alatawi F. S., Monier M., Elsayed N. H. (2018). Amino functionalization of carboxymethyl cellulose for efficient immobilization of urease. Int. J. Biol. Macromol..

[cit58] Shaw W. H., Raval D. N. (1961). The inhibition of urease by methylurea. J. Am. Chem. Soc..

[cit59] Uesato S., Hashimoto Y., Nishino M., Nagaoka Y., Kuwajima H. (2002). N-substituted hydroxyureas as urease inhibitors. Chem. Pharm. Bull..

[cit60] Sivapriya K., Suguna P., Banerjee A., Saravanan V., Rao D. N., Chandrasekaran S. (2007). Facile one-pot synthesis of thio and selenourea derivatives: A new class of potent urease inhibitors. Bioorg. Med. Chem. Lett..

[cit61] Perveen S. , Process for the preparation of" Urchym" a urease and alpha-chymotrypsin enzyme inhibitory drug, US Pat., 2008/0221214 A1, 2008

[cit62] Kappaun K., Piovesan A. R., Carlini C. R., Ligabue-Braun R. (2018). Ureases: Historical aspects, catalytic, and non-catalytic properties–A review. J. Adv. Res..

[cit63] Krajewska B., Piwowarska Z. (2005). Free vs. chitosan-immobilized urease: Microenvironmental effects on enzyme inhibitions. Biocatal. Biotransform..

[cit64] Mondal H., Datta B. (2023). Banana Peel Derived Chitosan-Grafted Biocomposite for Recovery of NH4+ and PO_4_^3–^. ACS Omega.

