# Supplementary material for: Ammonium release in synthetic and human urine by a urease immobilized nanoconstruct
Source: RSC Adv. 2024 Feb 27;14(10):6972–84. doi: 10.1039/d3ra07606g (PMC10898436; doi:10.1039/d3ra07606g)
Supplement: RA-014-D3RA07606G-s001 [file RA-014-D3RA07606G-s001.pdf]

Electronic Supplementary Material (ESI) for RSC Advances.
This journal is © The Royal Society of Chemistry 2024

Supporting Information

Ammonium Release in Synthetic and Human Urine by Urease Immobilized Nanoconstruct

Manab Diasi[1, ‡], Rinki Singh[1, ‡,*], Amarjyoti Das Mahapatra[1], Renuka L[1], Hitarth Patel[1], Hasit Ganatra[2], Bhaskar Datta[1,3,*]

[1]Department of Chemistry, Indian Institute of Technology Gandhinagar, Palaj, Gandhinagar - 382355, Gujarat, India

[2]Blasto Research Private Limited, Ahmedabad, Gujarat, India.

[3]Department of Biological Engineering, Indian Institute of Technology Gandhinagar, Palaj, Gandhinagar - 382355, Gujarat, India

[‡]These authors contributed equally to the work

* Corresponding author.

E-mail: bdatta@iitgn.ac.in; rinkoosingh62@gmail.com

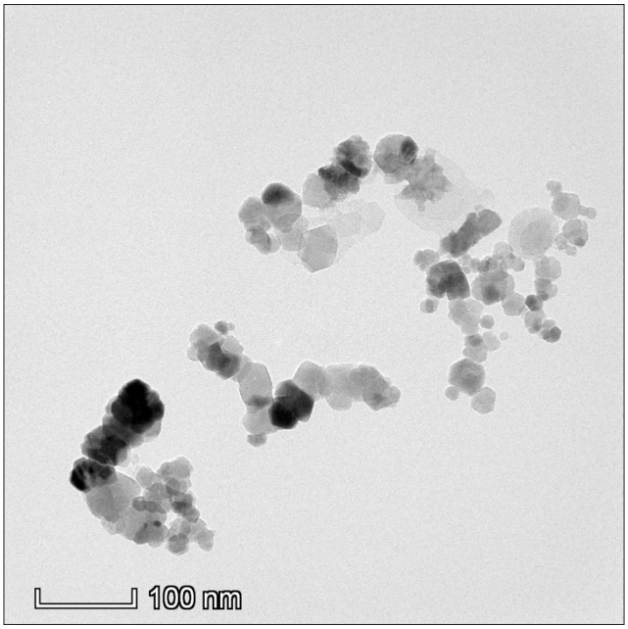

Figure S1: TEM image of MIONPs (Fe_3O_4 nanoparticles)

Figure S1, represents the TEM image of MIONPs (Fe_3O_4 nanoparticles). The morphology and size of MIONPs were observed by transmission electron microscope (FEI Themis 60−300 with an EDS detector and an FEI-Ceta 4k*4k camera) operating at an acceleration voltage of 200 kV. As shown in Figure S1, the observed average size of nanoparticles was about 15-30 nm, which was consistent with the X-ray diffraction (XRD) result, where the particle size was estimated using Debye-Scherrer expression (as described in the main Manuscript, Section 3.1).

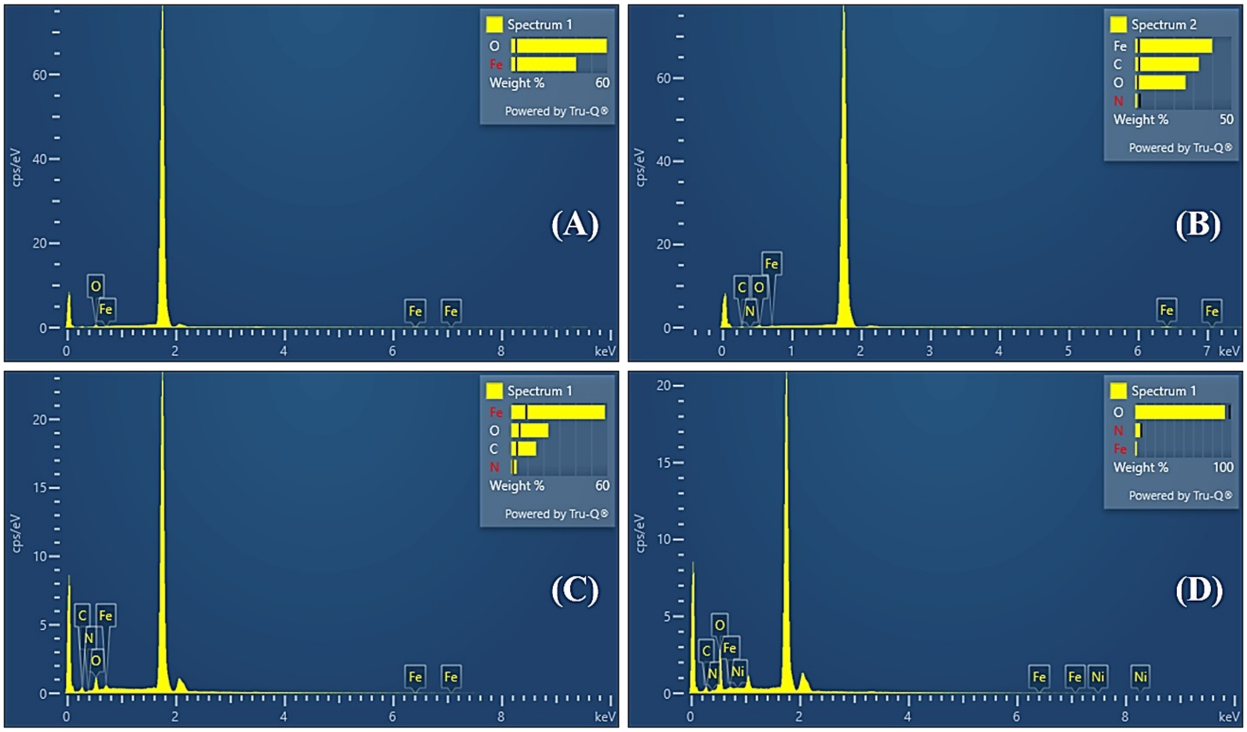

Figure S2: EDAX spectra of (A) MIONPs, (B) CS/MIONPs, (C) GA/CS/MIONPs and (D) urease/GA/CS/MIONPs

Figure S2, shows the EDAX spectra of MIONPs, CS/MIONPs, GA/CS/MIONPs and urease/GA/CS/MIONPs. The spectrum of bare MIONPs contained four peaks, which are assigned to Fe and O. However, CS/MIONPs and GA/CS/MIONPs contained six peaks that assigned not only for Fe and O but also C, N and O. Furthermore, the peak of Ni exhibits the existence of urease in urease/ GA/CS/MIONPs.

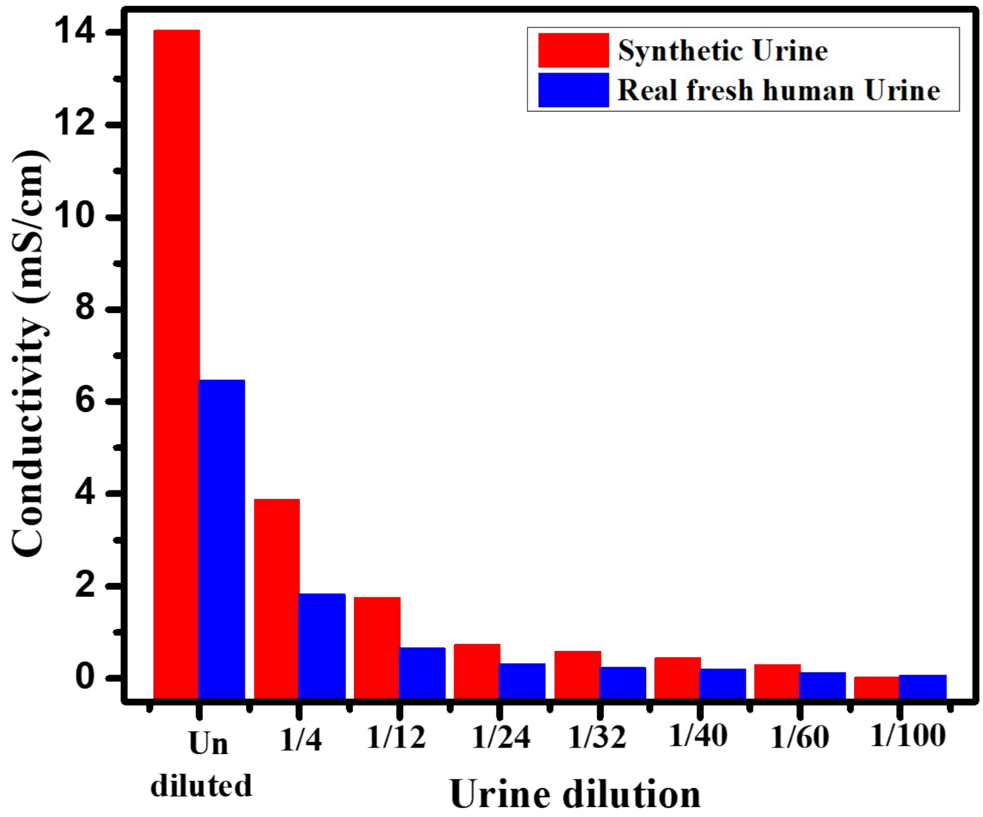

Figure S3: Effect of dilution of synthetic and real fresh human urine on urease nano construct by ammonia generation in terms of conductivity measurements.

Figure S3, shows the effect of dilution of synthetic and real fresh human urine on urease nano construct by ammonia generation in terms of conductivity measurements. Since the hydrolysis reaction results in the production of ammonium with the dilution of urine solutions, it was predicted that the conductivity, a measurement of the total current-carrying capacity of each ion in solution, would decrease over time[1].

Table S1. Characteristics obtained in the DLS determinations for pristine MIONPs, CS/MIONPs, GA/CS/MIONPs and Urease/GA/CS/MIONPs

Sample	Hydrodynamic Size (D_h) in nm
MIONPs	187.7 ± 5.2
CS/MIONPs	257.5 ± 4.9
GA/CS/MIONPs	1485 ± 5.9
Urease/GA/CS/MIONPs	973.1 ± 6.7

Table S1, shows the hydrodynamic size of pristine MIONPs, CS/MIONPs, GA/CS/MIONPs and Urease/GA/CS/MIONPs were obtained using dynamic light scattering (DLS).

Preparation of Synthetic Urine (SUr)

Water (95% by volume) makes up most of human urine. Urea (2%), creatinine (0.1%), uric acid (0.03%), chloride, sodium, potassium, sulphate, ammonium, phosphate, and other ions and molecules are included in the remaining components[2]. For a straightforward and cost-effective SUr formulation, the number of constituents is maintained to a minimum. Only the elements with a significantly larger concentration are therefore preferred. Table S2 contains a list of the SUr components employed in the current investigation along with their final concentrations. Using a magnetic stirrer moving between 250 and 500 rpm, the components are dissolved in 100 mL of double-distilled water. The solution's temperature is held at 37.5 °C throughout mixing by using the stirrer's heater function[3].

Table S2. The composition of synthetic urine (SUr) used in this study

Component	Molarity (mM)	Quantity (g/100ml)
Na_2SO_4	11.965	0.1700
Uric Acid ($C_5H_4N_4O_3$)	1.487	0.0250
Sodium Citrate ($Na_3C_6H_5O_7.2H_2O$)	2.450	0.0720
Creatinine ($C_4H_7N_3O$)	7.791	0.0881
Urea (CH_4N_2O)	249.750	1.5000
KCl	30.953	0.2308
NaCl	30.053	0.1756
$CaCl_2$	1.663	0.0185
Ammonium Chloride (NH_4Cl)	23.667	0.1266
Potassium Oxalate ($K_2C_2O_4.H_2O$)	0.19	0.0035
$MgSO_4.7H_2O$	4.389	0.1082
$NaH_2PO_4.2H_2O$	18.667	0.2912
$Na_2HPO_4.2H_2O$	4.667	0.0831

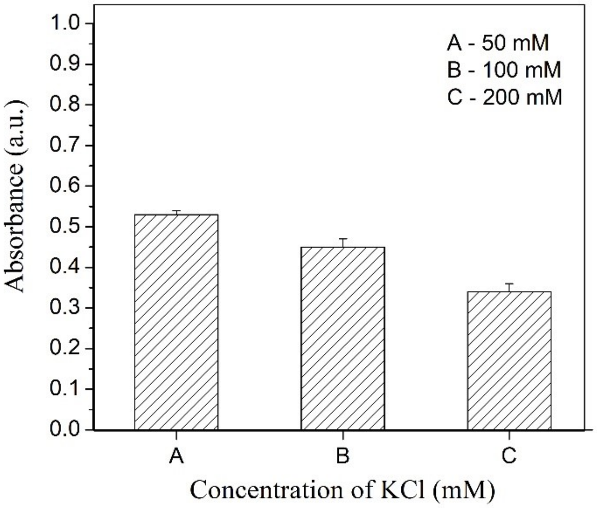

Figure S4: Effect of KCl concentration on urease-GA/CS/MIONP activity.

Figure S4, shows the effect of three different KCl concentrations on urease-GA/CS/MIONP facilitated ammonium production as measured by Berthelot reaction. Each sample (A-C) contained identical amounts of urea and ammonium chloride as those used in SUr, and in addition only the amount of KCl indicated. Experiments were performed in an identical manner as those of Urease/GA/CS/MIONP on SUr.

Table S3. Ammonium ion concentration corresponding to SUr and HUr treated with Urease/GA/CS/MIONPs

	NH_4^+ (mg/L)	
**Urine Type and Dilution**	**-Urease/GA/CS/MIONPs**	**+Urease/GA/CS/MIONPs**
SUr, 1/20	2.65	7.81
SUr, 1/24	2.26	7.86
HUr, 1/20	2.98	7.65
HUr, 1/24	2.88	7.86

Table S4. Comparison of k_m and v_{max} values of Urease/GA/CS/MIONPs calculated from the Lineweaver-Burk plots of the Michalis-Menten from other reported literature.

**Urease immobilized support**	k_m**(mM)**	v_{max} **(µM min^{-1})**
[4]Graphene oxide-chitosan immobilized urease	9.14	--
[5]Amino-functionalised carboxy methyl cellulose beads immobilized urease	14	--
[6]Chitosan nanoparticles immobilized urease	11.36	63.69
[7]p(Aam-co-AA)/ carrageenan immobilized urease	--	9.9
[8]Nylon membrane immobilized urease	18.1 (soluble urease)<br>13.9 (insoluble urease)	11.5<br>10.8
[9]Polymer phosphine oxide support urease	15	--
[10]Cotton fibres immobilized urease	9.54	22.44
Urease/GA/CS/MIONPs (Current Study)	14.86 (Synthetic Urine, SUr)<br>12.36 (Human Urine, HUr)	18.55 (Synthetic Urine, SUr)<br>10.10 (Human Urine, HUr)

References

1. Ray, H.; Saetta, D.; Boyer, T. H., Characterization of urea hydrolysis in fresh human urine and inhibition by chemical addition. *Environmental Science: Water Research & Technology* **2018,** 4, (1), 87-98.
2. Putnam, D. F. *Composition and concentrative properties of human urine*; NASA: 1971.
3. Sarigul, N.; Korkmaz, F.; Kurultak, İ., A new artificial urine protocol to better imitate human urine. *Scientific reports* **2019,** 9, (1), 1-11.
4. Yang, L.; Liu, X.; Zhou, N.; Tian, Y., Characteristics of refold acid urease immobilized covalently by graphene oxide-chitosan composite beads. *Journal of bioscience and bioengineering* **2019,** 127, (1), 16-22.
5. Alatawi, F. S.; Monier, M.; Elsayed, N. H., Amino functionalization of carboxymethyl cellulose for efficient immobilization of urease. *International journal of biological macromolecules* **2018,** 114, 1018-1025.
6. Al-Garawi, Z. S.; Taha, A. A.; Abd, A. N.; Tahir, N. T., Immobilization of urease onto nanochitosan enhanced the enzyme efficiency: biophysical studies and in vitro clinical application on nephropathy diabetic iraqi patients. *Journal of Nanotechnology* **2022,** 2022.
7. Kara, F.; Demirel, G.; Tümtürk, H., Immobilization of urease by using chitosan–alginate and poly (acrylamide-co-acrylic acid)/κ-carrageenan supports. *Bioprocess and biosystems engineering* **2006,** 29, 207-211.
8. Di Martino, S.; El-Sheriff, H.; Diano, N.; De Maio, A.; Grano, V.; Rossi, S.; Bencivenga, U.; Mattei, A.; Mita, D., Urea removal from agricultural waste waters by means of urease immobilized on nylon membranes grafted with cyclohexyl-methacrylate. *Applied Catalysis B: Environmental* **2003,** 46, (3), 613-629.
9. Sarfo, K.; Petach, H. H.; Henderson, W., Polymeric phosphine oxide polyether-derived copolymer as a support for urease immobilization. *Enzyme and microbial technology* **1995,** 17, (9), 804-808.
10. Monier, M.; El-Sokkary, A., Modification and characterization of cellulosic cotton fibers for efficient immobilization of urease. *International journal of biological macromolecules* **2012,** 51, (1-2), 18-24.
